# Supplementary material for: Discrete populations of isotype-switched memory B lymphocytes are maintained in murine spleen and bone marrow
Source: Nat Commun. 2020 May 22;11:2570. doi: 10.1038/s41467-020-16464-6 (PMC7244721; doi:10.1038/s41467-020-16464-6)
Supplement: Supplementary file 1 — Supplementary Information [file 41467_2020_16464_MOESM1_ESM.pdf]

Supplementary Information

Discrete populations of isotype-switched memory B lymphocytes are maintained in murine spleen and bone marrow

Riedel et al.

Supplementary Figure 1

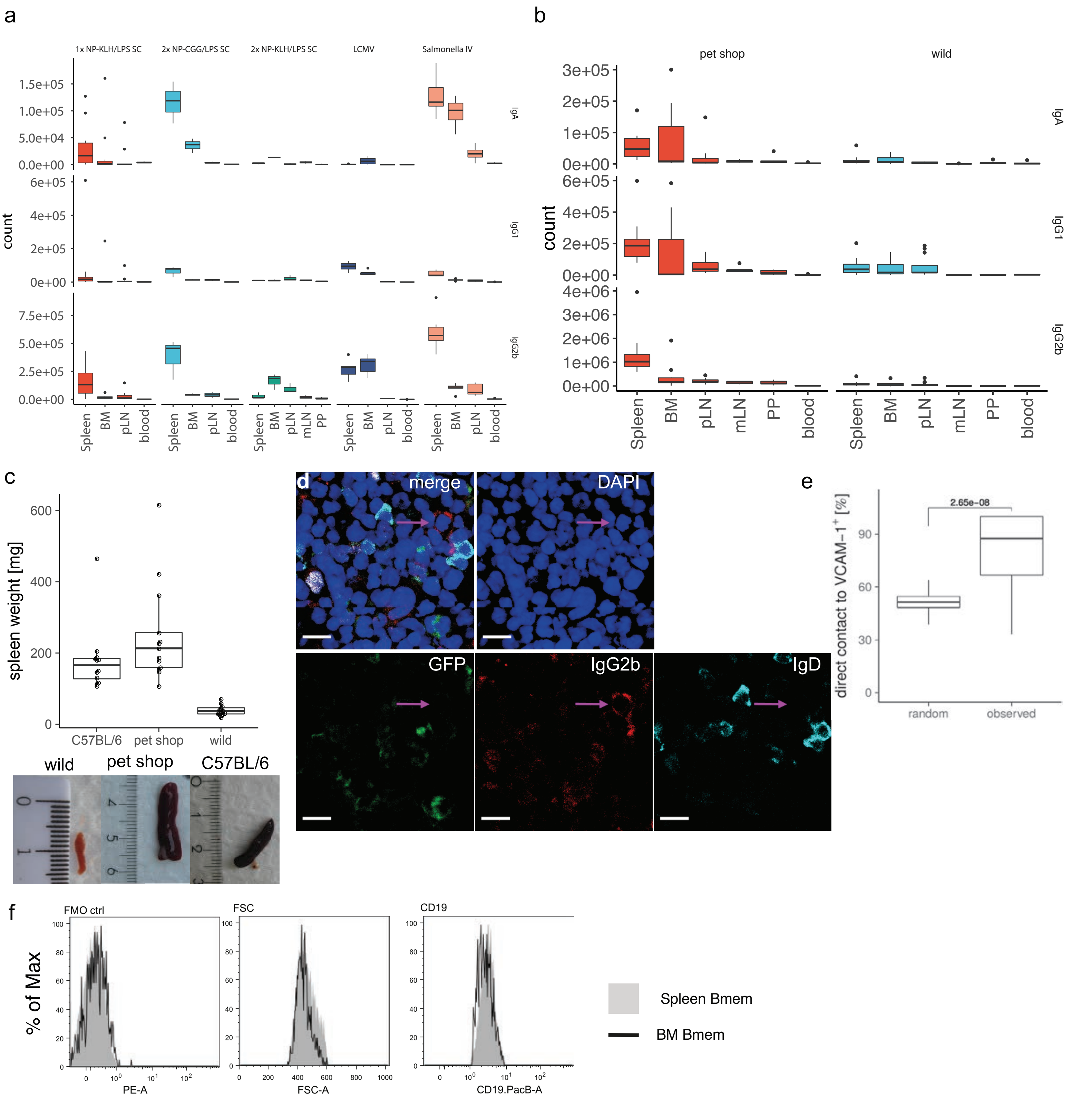

Supplementary Figure 1: a) Cell numbers of Ig-switched memory B cells per organ in C57BL/6 laboratory mice. Absolute cell numbers per organ calculated from flow cytometric counts (gated for IgG1+, IgG2b+, or IgA+ CD19+CD38+CD138-GL7-CD11c-IgM-IgD-PI- small lymphocytes); n=42, data pooled from 8 experiments with five different immunizations protocols performed in C57BL/6 mice aged 4-20 months and held under SPF conditions, data presented as median cell count per organ by immunization. BM: bone marrow, pLN: peripheral lymph nodes, mLN: mesenteric lymph nodes, PP: Peyer's patches. b) Cell numbers of Bsm per organ in wild and pet shop mice. Median cell numbers per organ calculated from flow cytometric counts (gated for IgG1+, IgG2b+, or IgA+ CD19+CD38+CD138-GL7-CD11c-IgM-IgD-PI- small lymphocytes); n=13 (wild) and 13 (pet). c) Spleen weight from spleens of C57BL/6 mice held under SPF conditions, pet shop and wild mice, n=10 (C57BL/6), 13 (pet), and 11 (wild). Boxplots indicate median, first and third quartiles, whiskers: 1.5 IQR (c-g). d) Identification of BM IgG2b+ Bsm. Naive B cells and plasma cells were excluded by IgD staining and Blimp1-GFP signal, respectively. Cells were identified by nuclei (DAPI, blue). Scale bar: 10μm. Representative micrograph of identification strategy used for quantification of colocalization of Bsm with other cell types. e) Simulation of co-localization between Bsm and VCAM-1+ stromal cells. Non-random co-localization of BM Bsm was determined using images acquired from 7 bone marrow slides. Graphs represent direct co-localization of more than 12000 simulated cells (random) versus co-localization observed per slide for 28 slides from 4 mice with two or more analyzed Bsm per slide (mean=5.66 cells per slide), p value (Welch's test, one-sided) indicated on graph. f) Controls (CD19, PE-FMO: fluorescence minus one control PE, FSC-A) for spleen and BM memory B cells in flow cytometry, identified as IgG2b+CD19+CD38+CD138-GL7-CD11c-IgM-IgD-PI- small lymphocytes.

Supplementary Figure 2

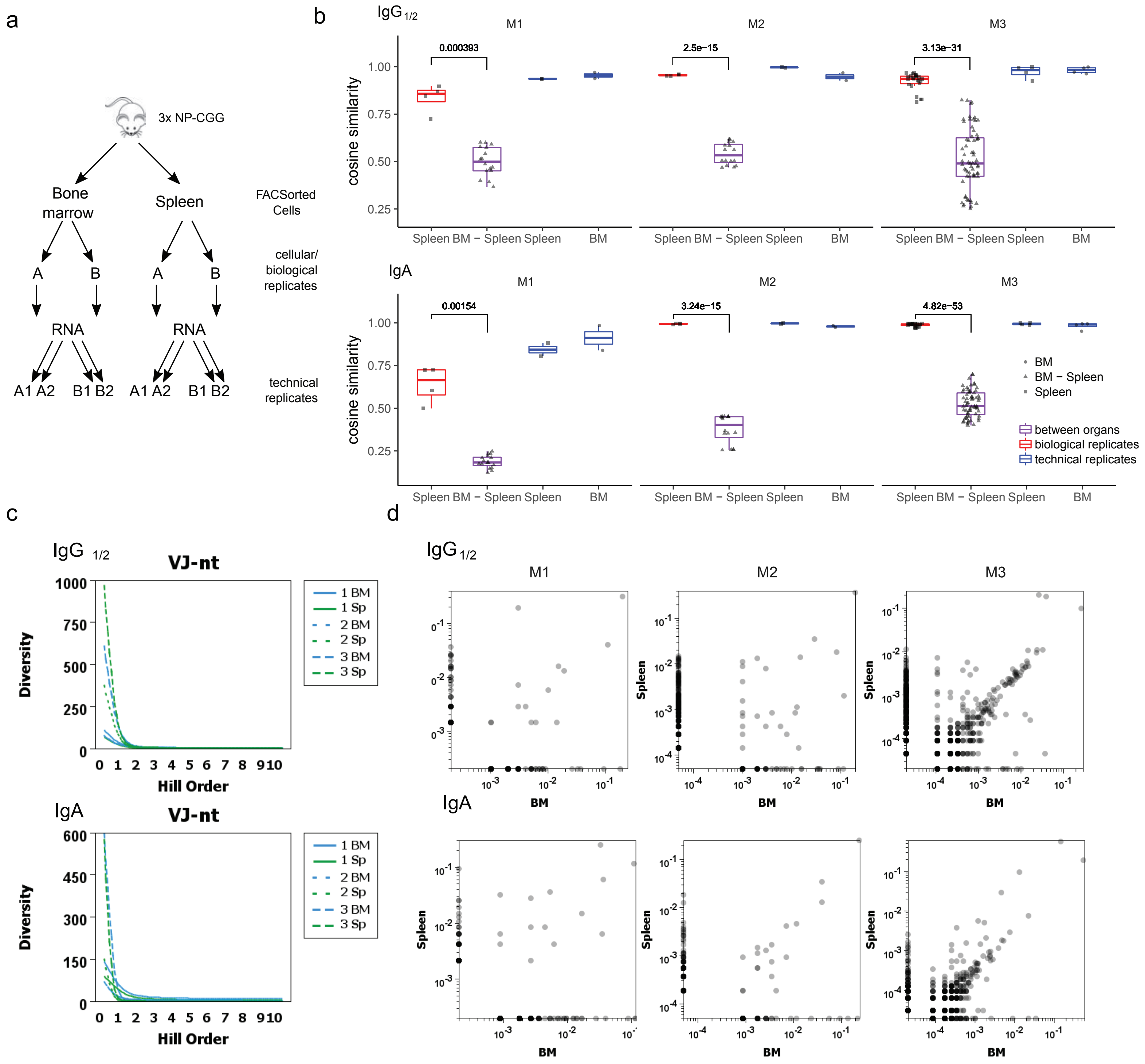

Supplementary Figure 2: a) Experimental setup for the comparison of repertoire of isotype-switched memory B cells of BM and spleen: after isolation, cells of the same organ were divided into equal proportions and processed as biological replicates. After RNA isolation, samples were split and processed as technical replicates. b) Cosine similarity between samples of Ig heavy chain CDR3 repertoires accounting for clonotype frequencies. Graphs represent cosine similarity comparison within technical replicates of spleen and BM (blue), within cellular replicates from spleens (red), and between spleen and BM (BM-Spleen, purple) of three individual mice. p values (one-sided Welch's test) for difference of means of cosine similarity within shared IgH repertoire (spleen cellular replicates) and between spleen and BM replicates are indicated. Boxplots indicate median, first and third quartiles, whiskers: 1.5 IQR. c) Clonotype diversity within spleen and BM of three individual mice represented at different Hill orders, where Hill order of 0 represents the observed number of different clonotypes. d) Clonotype frequency as observed in spleen (Spleen) and BM of three individual mice. Data points mapped directly on the axes represent clonotypes present only in the respective organ. Clonotypes were defined by VH and JH genes in combination with CDR3H nucleotide sequence, upper panels IgG1/2, lower panels IgA (b-d).

Supplementary Figure 3

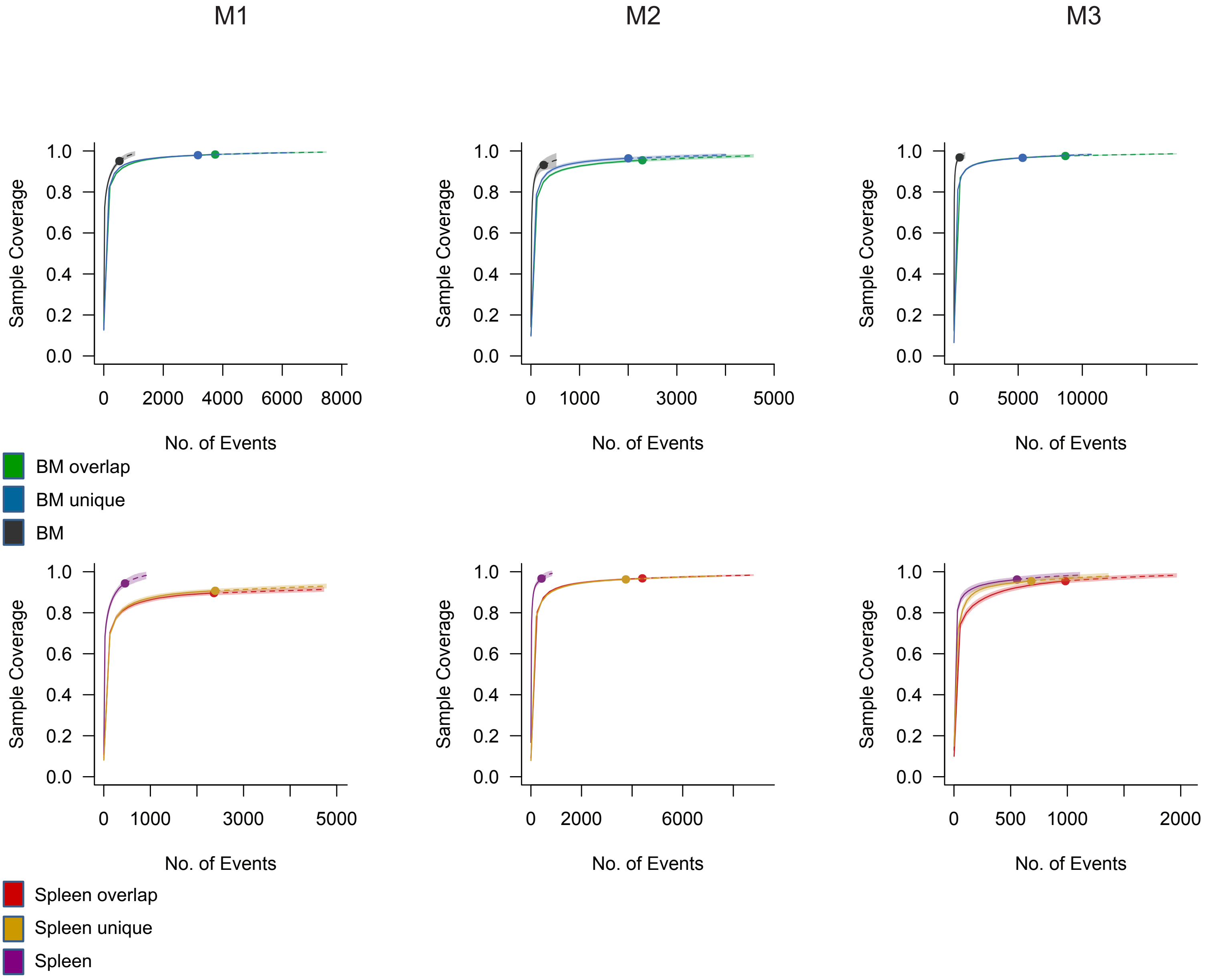

Supplementary Figure 3: Estimated sample completeness, based on rarefaction (solid lines) and extrapolation (dashed lines) with increasing sequencing depth for bone marrow (BM, upper panel) and spleen for all shared (overlap, present in spleen and BM) and exclusive clonotypes to the respective organ (unique). Dots indicate the actual sequencing depth and the corresponding sample completness of clonotypes in a sample. 95% confidence intervals are shaded.

Supplementary Figure 4

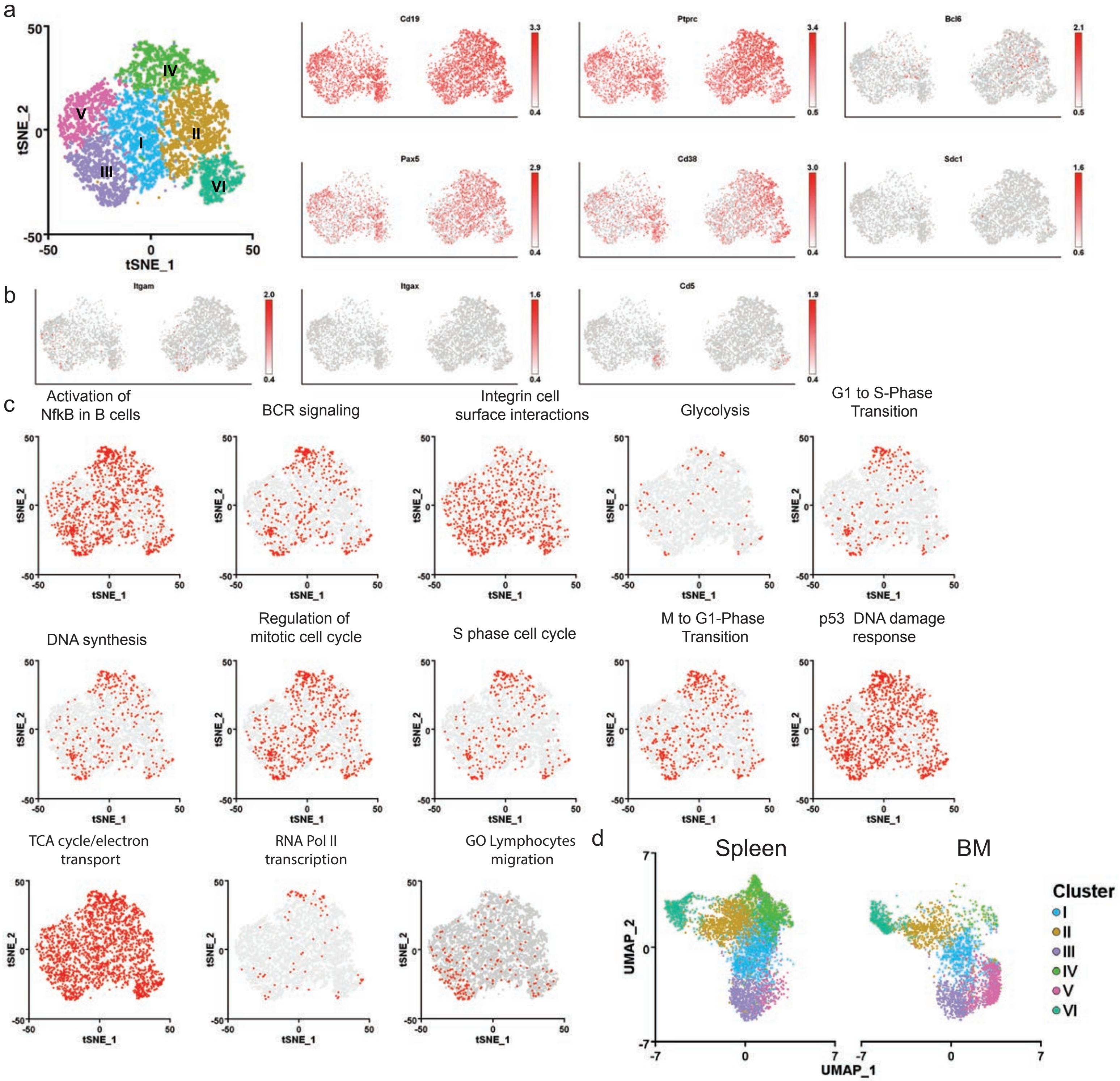

Supplementary Figure 4: a) Six transcriptionally defined Clusters were identified by shared nearest neighbor (SNN) modularity optimization based clustering algorithm mapped to tSNE representation of spleen and bone marrow (BM) cells. tSNE coordinates and clustering was computed for 4754 from spleen and 2947 from BM cells, representation combined for spleen and BM (left). Distribution of expression for Cd19, Ptprc (CD45), Pax5, Cd38, Sdc1 (CD138), and Bcl6 genes mapped on tSNE representation. b) Distribution of expression for Itgax (CD11c), Itgam (CD11b), Cd5 genes mapped on tSNE representation. c) Single cell gene set enrichment analysis of expression of the Reactome and Gene ontology enrichment (GO) mapped on tSNE representation. Cells for single cell sequencing were FACSorted as IgG-expressing CD19+CD38+CD138-GL7- small lymphocytes. d) Six transcriptionally defined clusters were identified by shared nearest neighbor (SNN) modularity optimization based clustering algorithm mapped to UMAP representation of spleen and BM cells. UMAP coordinates and clustering was computed for 4754 from spleen and 2947 from BM cells, presentation is separated by organ.

Supplementary Figure 5

a

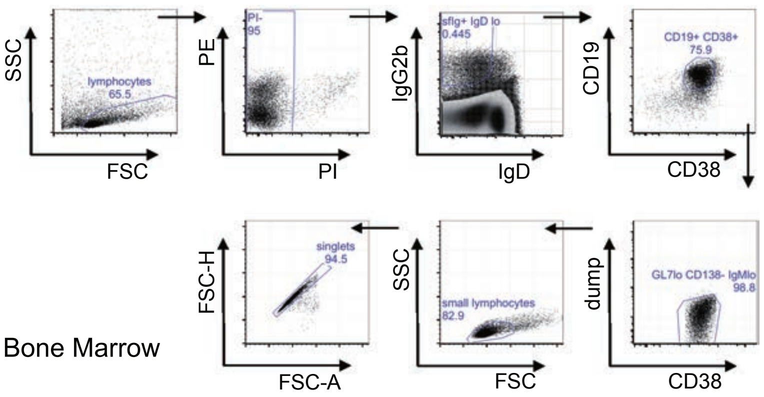

b

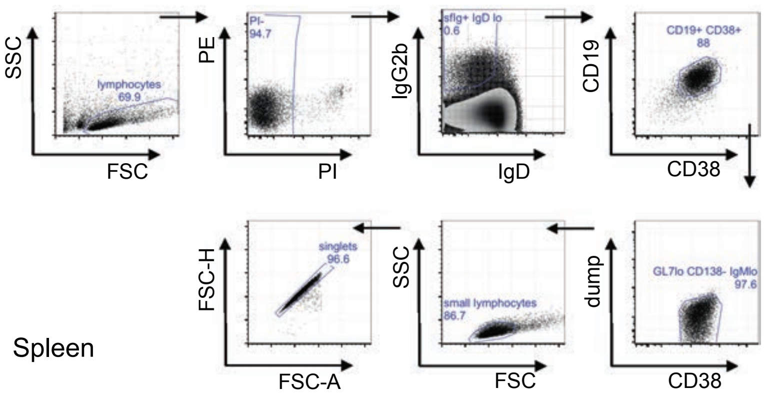

c

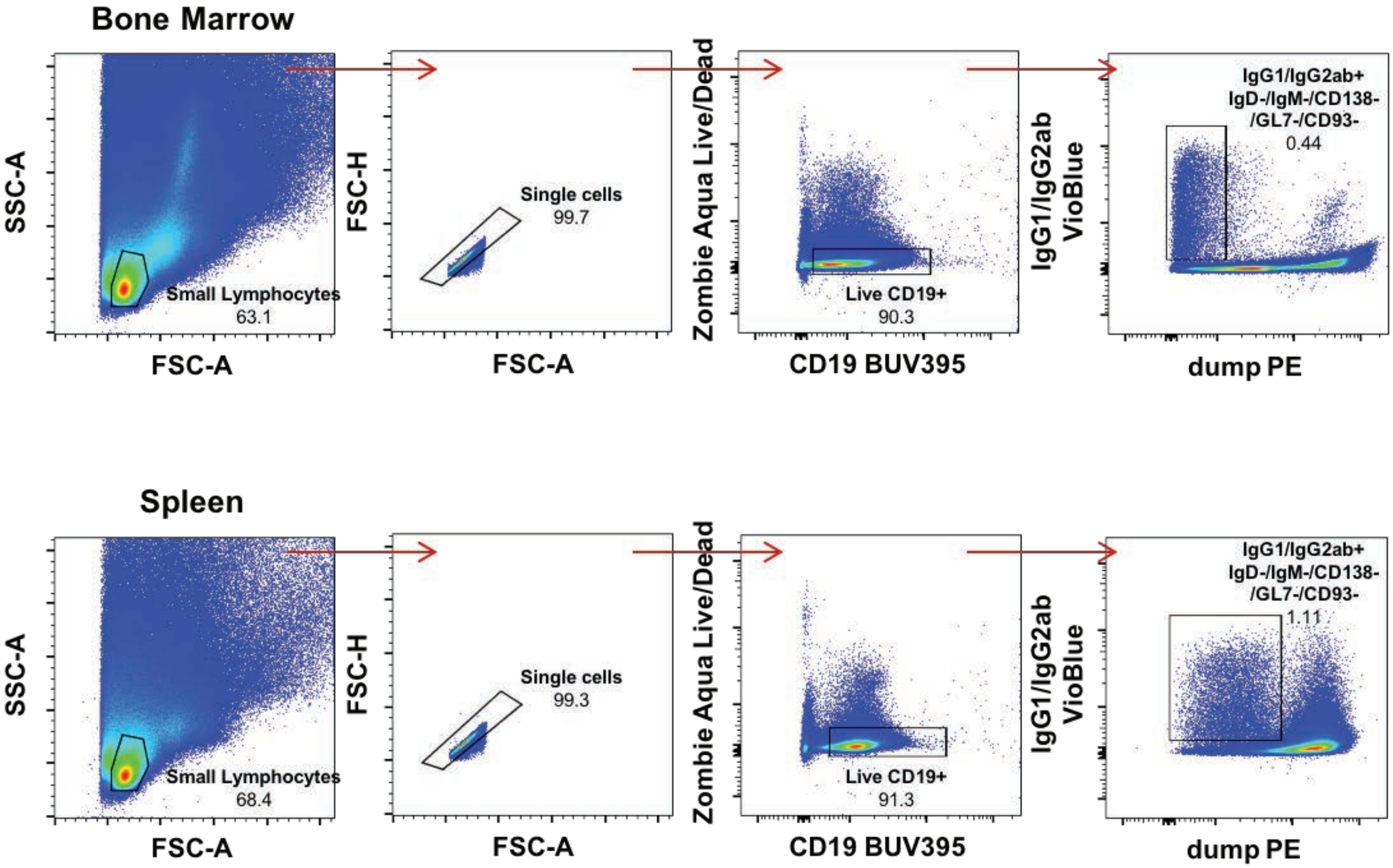

Supplementary Figure 5: Gating for isotype-switched memory B cells of bone marrow and spleen. BM (a) or spleen (b) CD19+ cells were isolated by MACS technology and switched-memory B cells were identified by expression of surface IgG2b, IgG1, or IgA and CD19, CD38 and lack of IgD, IgM, CD138 and GL7 marker. Staining shown for IgG2b exemplarily. Gating strategy used for analysis presented in Figure 1a-c,g. c) Gating for isotype-switched memory B cells of bone marrow and spleen. BM or spleen CD19+ cells were isolated by MACS technology and switched-memory B cells were identified as small lymphocytes and by expression of surface IgG2b, IgG1 and CD19, CD38 and lack of IgD, IgM, CD138, CD93 and GL7 marker. Gating strategy used for analysis presented in Figure 4a-c.

Supplementary Table 1: *Diversity metrics for bulk and single cell Ig repertoire analyses. Count: number of sequences (bulk) or cells (single cell), Diversity: observed number of unique clonotypes, Evenness: Shannon's evenness, Simpson: Simpson diversity, Chao1: estimated species richness (diversity estimate).*

Experiment	Sample	Isotype	Count	Diversity	Evenness	Simpson	Chao1
**bulk**	M1 BM	$IgG_{1/2}$	983	113	0.663	0.899	154
	M1 Spleen		682	71	0.654	0.850	104
	M2 BM		997	79	0.688	0.911	119
	M2 Spleen		6911	376	0.629	0.854	491
	M3 BM		9051	610	0.636	0.916	955
	M3 Spleen		22189	967	0.595	0.911	1,372
	M1 BM	IgA	1117	144	0.731	0.942	195
	M1 Spleen		464	91	0.718	0.904	179
	M2 BM		1124	72	0.584	0.857	107
	M2 Spleen		5271	152	0.230	0.329	231
	M3 BM		11535	596	0.373	0.662	957
	M3 Spleen		30638	575	0.277	0.624	782
**single cell**	M1 BM	NA	2059	1368	0.911	0.988	8,478
	M1 Spl		2393	1863	0.953	0.996	1,195
	M2 BM		888	676	0.971	0.997	3,718
	M2 Spl		2358	1928	0.983	0.999	12,230
