# Supplementary material for: Discrete populations of isotype-switched memory B lymphocytes are maintained in murine spleen and bone marrow
Source: Nat Commun. 2020 May 22;11:2570. doi: 10.1038/s41467-020-16464-6 (PMC7244721; doi:10.1038/s41467-020-16464-6)
Supplement: Supplementary file 3 — Reporting Summary [file 41467_2020_16464_MOESM3_ESM.pdf]

nature research

Corresponding author(s): Mir-Farzin Mashreghi and Andreas Radbruch

Last updated by author(s): Apr 23, 2020

Reporting Summary

Nature Research wishes to improve the reproducibility of the work that we publish. This form provides structure for consistency and transparency in reporting. For further information on Nature Research policies, see Authors & Referees and the Editorial Policy Checklist.

Statistics

For all statistical analyses, confirm that the following items are present in the figure legend, table legend, main text, or Methods section.

n/a	Confirmed	
☐	☒	The exact sample size (*n*) for each experimental group/condition, given as a discrete number and unit of measurement
☐	☒	A statement on whether measurements were taken from distinct samples or whether the same sample was measured repeatedly
☐	☒	The statistical test(s) used AND whether they are one- or two-sided<br>*Only common tests should be described solely by name; describe more complex techniques in the Methods section.*
☐	☒	A description of all covariates tested
☐	☒	A description of any assumptions or corrections, such as tests of normality and adjustment for multiple comparisons
☐	☒	A full description of the statistical parameters including central tendency (e.g. means) or other basic estimates (e.g. regression coefficient) AND variation (e.g. standard deviation) or associated estimates of uncertainty (e.g. confidence intervals)
☐	☒	For null hypothesis testing, the test statistic (e.g. *F*, *t*, *r*) with confidence intervals, effect sizes, degrees of freedom and *P* value noted<br>*Give P values as exact values whenever suitable.*
☒	☐	For Bayesian analysis, information on the choice of priors and Markov chain Monte Carlo settings
☐	☒	For hierarchical and complex designs, identification of the appropriate level for tests and full reporting of outcomes
☐	☒	Estimates of effect sizes (e.g. Cohen's *d*, Pearson's *r*), indicating how they were calculated

Our web collection on statistics for biologists contains articles on many of the points above.

Software and code

Policy information about availability of computer code

Data collection	Raw sequencing data for single cell transcriptome analysis were processed using cellranger-2.1.1. mkfastq and count commands. Raw sequencing data for single cell immune profiling (B cell receptor sequences (BCR) for IgG heavy and light chain) were processed using cellranger-3.0.2. mkfastq and vdj commands. The high-confidence contig sequences of the isotype-switched memory B cells were reanalyzed using the HighV-QUEST database at IMGT web portal for immunoglobulin (IMGT 1.2.8 , database release LIGMDB_V12) to retrieve the V-, J- and D-genes as nucleotide and amino acid CDR3 sequence. Lineage trees were computed using GLaMST (w/o versioning). The single cell transcriptome data was further analyzed using R 3.5.0., and Seurat R package 2.3.4. Bulk Ig repertoire analysis was performed using MIGEC-1.2.4a.
Data analysis	Statistics and data analysis was performed in R (version 3.5). Additionally analyses were performed using Java. Modelling of random cell positioning, in a modification of the previously published approach (Zehentmeier et al, Eur J Immunol 2014.).<br>Code will be made available on request.

For manuscripts utilizing custom algorithms or software that are central to the research but not yet described in published literature, software must be made available to editors/reviewers. We strongly encourage code deposition in a community repository (e.g. GitHub). See the Nature Research guidelines for submitting code & software for further information.

Data

Policy information about availability of data

All manuscripts must include a data availability statement. This statement should provide the following information, where applicable:

- Accession codes, unique identifiers, or web links for publicly available datasets
- A list of figures that have associated raw data
- A description of any restrictions on data availability

Sequencing data have been deposited at GEO (https://www.ncbi.nlm.nih.gov/geo/; superseries GSE140133).
Cytometry and histological data will be made vailable by the authors upon reasonable request.

Field-specific reporting

Please select the one below that is the best fit for your research. If you are not sure, read the appropriate sections before making your selection.

☒ Life sciences ☐ Behavioural & social sciences ☐ Ecological, evolutionary & environmental sciences

For a reference copy of the document with all sections, see nature.com/documents/nr-reporting-summary-flat.pdf

Life sciences study design

All studies must disclose on these points even when the disclosure is negative.

Sample size	Sample size was determined on the basis of expected effect size. In brief, t-tests with simulated data was performed for experiments for figures 1a-c . Data for sequencing was performed with minimal sample size of 3 (bulk receptor repertoires) and 2 (single cell RNA and Ig repertoire).<br>Data collection for lab mice from different immunizations (Suppl. Fig1a) was performed by sample sharing of mice used for other experiments. Sample collection from wild mice was part of pest control measures and performed over the time period licenced, number of pet mice was chosen accordingly. No sample size calculations were performed prior to data collection.
Data exclusions	No data were excluded during analysis.
Replication	Quantification of antigen-specific memory B cells of mice and for survival of memory B cells during Cyclophosphamide treatment were pooled from 2 independent experiments each. Quantification of switched memory B cells was obtained from mice of various different backgrounds to ascertain general validity of findings.<br>Preferential co-lozalisation of cells in histology was tested against random distribution 1000 times. Difference in distribution of BCR clonotypes to spleen and bone marrow or to specific populations within those were tested against random distribution of observed sequences 1000 times. Distribution of clonally related BCRs to different subsets was tested against random distribution 1000 times.<br>All attempts to replication of results was successful.
Randomization	Study design did not necessitate randomization, internal controls were used as appropriate (i.e. comparing Bsm of different organs or different populations within the same animals, blocking antigen specific staining). To confirm non-random distribution of cells in situ or BCR clones, the statistical significance of observed vs simulated random distribution was determined.
Blinding	Blinding was not performed, since mostly paired samples of the same animals had to be compared due to experimental design.

Reporting for specific materials, systems and methods

We require information from authors about some types of materials, experimental systems and methods used in many studies. Here, indicate whether each material, system or method listed is relevant to your study. If you are not sure if a list item applies to your research, read the appropriate section before selecting a response.

Materials & experimental systems

n/a	Involved in the study	
☐	☒	Antibodies
☒	☐	Eukaryotic cell lines
☒	☐	Palaeontology
☐	☒	Animals and other organisms
☒	☐	Human research participants
☒	☐	Clinical data

Methods

n/a	Involved in the study	
☒	☐	ChIP-seq
☐	☒	Flow cytometry
☒	☐	MRI-based neuroimaging

Antibodies

Antibodies used	For cytometry: CD5 (53-7.3, BV786, BD OptiBuild Cat No 740842, 1:200), CD11b (M1/70, BV650, BioLegend Cat No 101239, 1:25), CD19 (1D3, PacB DRFZ, and 1D3, BUV395, BD Horizon Cat No 563557, 1:400), CD21/35 (76G, BUV737, BioLegend, Cat No 62810, 1:400, and 7E9, APC-Cy7, BioLegend Cat No 123418, 1:400), CD23 (B3B4, PE-Cy7, BioLegend, Cat No 101614, 1:400), CD29 (HMß1 1, PE, Miltenyi Cat No 130-102-602, 1:25), CD38 (90, PE-cy7, BioLegend Cat No 102718, 1:400), CD49d (R1-2, PE, BioLegend Cat No 103608, 1:200 and REA807, VioBright515, Miltenyi Cat No 130-111-829, 1:200), CD49f (GoH3, PE, eBioscience Cat No 12-0495-82, 1:100), CD93 (REA298, PE, Miltenyi Cat No 130-104-157, 1:50, CD138 (281-2, PE, BD Biosciences Cat No 553714, 1:800), CXCR3 (CXCR3-173, BV605, BioLegend Cat No 126523, 1:23), GL7 (GL7, PE, DRFZ), IgA (C10-3, FITC, BD Biosciences Cat No 559354, 1:100), IgD (11.26c, PE, AF430, DRFZ and REA772, PE, Miltenyi Cat No 130-111-310, 1:100), IgG1 (A85-1, FITC, BD Biosciences Cat No 553443, 1:1000, and X-56, VioBlue, Miltenyi Cat No 130-099-089, 1:50), IgG2a/b (R2-40, FITC 1:100, PE, 1:1000, BD Biosciences Cat No 553399 and X-57, VioBlue, Miltenyi Cat No 130-099-083, 1:50), IgG2b (A95-1, FITC, BD Biosciences Cat No 553988, 1:800 and MRG2b-1, FITC, BioLegend Cat No 406706, 1:1000), IgM (M41, AF405, DRFZ and REA979, PE, Miltenyi Cat No 130-116-209, 1:100) and Ki-67 (B56, PE, BD Biosciences Cat No 556027, 1:400). Dead cells were excluded using PI staining or the Zombie Aqua Fixable Viability Kit (BioLegend).

For Histology: anti-mouse IgG2b-AlexaFluor546 (RMG2b-1, BioLegend Cat No 406702 ,1:100); anti-GFP-AlexaFluor488 (rabbit polyclonal, Life Technologies Cat No A-21311, 1:100; rat monoclonal FM264G, BioLegend Cat No 338008,1:100), anti-fibronectin (rabbit polyclonal, Sigma Aldrich Cat No F3648,1:100), biotinylated anti-mouse Ki67 (Sol-15, eBioscience Cat No 13-5698-82, 1:100), biotinylated anti-mouse VCAM-1 (429, eBioScience Cat No 13-1061-82, 1:100), anti-human/mouse cadherin 17 (rabbit polyclonal, R&D Cat No AF8524, 1:50), anti-mouse laminin (rabbit polyclonal, Sigma Aldrich L9393, 1:100), anti-mouse IgD-AlexaFluor647 (11.26c, DRFZ), anti-mouse Thy1-Alexa Fluor 594 (T24, DRFZ), anti-mouse B220-AlexaFluor594/647 (RA3.6B4, DRFZ), anti-mouse CD11c-AlexaFluor647 (N418, DRFZ), donkey anti-rabbit polyclonal IgG-AlexaFluor488/647 (Thermo Fisher Cat No A-21206,1:600), strepatavidin-AlexaFluor594/647 (Thermo Fisher S323561:1000).

Validation

CD5 (53-7.3 BD OptiBuild, [van Ewijk W, van Soest PL, van den Engh GJ. J Immunol. 1981; 127(6):2594-2604.]), CD11b (M1/70 BioLegend [Springer T, et al. 1978. Eur. J. Immunol. 8:539.]), CD19 (1D3 DRFZ, and 1D3 BD Horizon [Tedder TF, Zhou LJ, Engel P. Immunol Today. 1994; 15(9):437-442.]), CD21/35 (7E9 BioLegend [Boackle S, et al. 2001 Immunity 15:775.]), CD23 (B3B4, BioLegend [Rao M, et al. 1987. J. Immunol. 138:1845]), CD29 (HMß1 1 Miltenyi [Noto, K. et al. (1995) Int. Immunol. 7(5): 835-842]), CD38 (90, BioLegend [Howard M, et al. 1993. Science 262:1056.]), CD49d (R1-2, BioLegend [Ferguson TA, et al. 1993. J. Immunol. 150:1172., Lawson BR, et al. 2007. J. Immunol. 178:5366.] and REA807 Miltenyi [Berlin, C. et al. (1993) Cell 74(1): 185-195]), CD49f (GoH3 eBioscience [Lyubimova et al J Clin Invest. 2010 Feb;120(2):446-56.]), CD93 (REA298 Miltenyi [Petrenko, O. et al. (1999) Immunity 10(6): 691-700]) CD138 (281-2 BD Biosciences [Jalkanen M, Nguyen H, Rapraeger A, Kurn N, Bernfield M. J Cell Biol. 1985; 101(3):976-984.]), CXCR3 (CXCR3-173 BioLegend [Krug A, et al. 2002. J. Immunol. 169:6079.]), GL7 (GL7 DRFZ [Laszlo G, et al. 1993. J. Immunol. 150:5252.]), IgA (C10-3 BD Biosciences [Wrammert J, Källberg E, Agace WW, Leanderson T. Eur J Immunol. 2002; 32(1):97-103]), IgD (11.26c DRFZ [Jin, Nat Immunol. 2012 Nov;13(11):1101-9]), IgG1 (A85-1, FITC, BD Biosciences [manufacturer: The A85-1 antibody reacts specifically with mouse IgG1 of Igh-Ca and Igh-Cb haplotypes. It does not react with other Ig isotypes. Detection of surface immunoglobulin on B lymphoma cells has been demonstrated with the A85-1 monoclonal antibody. A suspension of pooled mouse IgG1 was used as the source of immunogen.]), IgG2a/b (R2-40 BD Biosciences [Benson MJ, Dillon SR, Castigli E, et al. Cutting edge: the dependence of plasma cells and independence of memory B cells on BAFF and APRIL.. J Immunol. 2008; 180(6):3655-9]), IgG2b (MRG2b- BioLegend [Feldman S, et al. 2015. J Immunol. 194:2380.]), IgM (M41 DRFZ [Duong J Immunol. 2011 Dec 1; 187(11): 5596–5605.]) and Ki-67 (B56 BD Biosciences [Bruno S, Crissman HA, Bauer KD, Darzynkiewicz Z. Exp Cell Res. 1991; 196(1):99-106.] and Sol-15 eBioscience [Pang Nat Immunol. 2013 Mar;14(3):246-53. doi: 10.1038/ni.2514.]), anti-GFP (rabbit polyclonal, Life Technologies [Fünfschilling J Comp Neurol. 2004 Dec 20;480(4):392-414.], rat monoconal FM264G BioLegend [Stephen LA, et al. 2018. Dev. Cell. 47(1):122-132.e4.]), anti-fibronectin (rabbit polyclonal, Sigma Aldrich [Manran Liu et. Al. 2010, Cancer research, 70(24)]), anti-mouse VCAM-1 (429, eBioScience [Baerenwaldt J Immunol. 2016 Mar 15;196(6):2561-71.]), anti-human/mouse cadherin 17 (rabbit polyclonal R&D, Detects mouse and human Cadherin17 in direct ELISAs and Western blots. In direct ELISAs, less then 10% crossreactivity with recombinant human Cadherin17), anti-mouse laminin (rabbit polyclonal, Sigma Aldrich [Caire et. Al, J Bone and Mineral Res 2019, 34(8)]), anti-mouse Thy1 (T24 DRFZ [Yan Bioconjug Chem. 1991 Jul-Aug;2(4):207-10.]), anti-mouse B220-AlexaFluor594/647 (RA3.6B2 DRFZ [Coffman RL. 1982. Immunol. Rev. 69:5.]), anti-mouse CD11c (N418 DRFZ [Metlay JP, et al. 1990. J. Exp. Med. 171:1753.]), donkey anti-rabbit polyclonal IgG (Thermo Fisher [Ganns Histol Histopathol. 2006 Jan;21(1):41-51.])

Animals and other organisms

Policy information about studies involving animals; ARRIVE guidelines recommended for reporting animal research

Laboratory animals

C57BL/6J/N mice were purchased from Charles River (Sulzfeld, Germany) or Janvier Labs (Le Genest-Saint-Isle, France). Mice expressing GFP under the control of the Prdm1 promoter (Blimp1-GFP) 43 were bred at the DRFZ animal facility. C57BL/6 and Blimp1-GFP mice were housed under specific pathogen-free conditions with 12h light/dark cycle at 18-21°C with food and water ad libitum. Pet mice were obtained as adult animals at pet shops in Berlin.

Wild animals

Feral mice (male and female of uncertain age) were caught as free-living animals as part of a pest control measure at non-residential farm buildings in Altlandsberg, Brandenburg, Germany. Live traps were placed and controlled by a trained veterinarian. Mice were anesthetized with isofluran prior to sacrifice and organ harvest.

Field-collected samples

The study did not involve laboratory experiments with field collected samples. Mice caught in the field were sacrificed on site and measurements were obtained on the day of sacrifice.

Ethics oversight

All animal experiments were performed according to institutional guidelines and licensed under German animal protection regulations by the Landesamt für Gesundheit und Soziales Berlin and Landesamt für Arbeitsschutz (pet and lab mice), Verbraucherschutz und Gesundheit Brandenburg (feral mice).

Note that full information on the approval of the study protocol must also be provided in the manuscript.

Flow Cytometry

Plots

Confirm that:

☒ The axis labels state the marker and fluorochrome used (e.g. CD4-FITC).

☒ The axis scales are clearly visible. Include numbers along axes only for bottom left plot of group (a 'group' is an analysis of identical markers).

☒ All plots are contour plots with outliers or pseudocolor plots.

☒ A numerical value for number of cells or percentage (with statistics) is provided.

Methodology

Sample preparation	In order to stain lymphocytes for multicolor flow cytometry, cells were resuspended in PBS with 0.5% BSA and 5µg per ml Fcmu receptor IIB-blocking antibody (DRFZ, clone 2.4G2) or 10µl of FcR Blocking Reagent (Miltenyi) per 10e7 cells at up to 5x10e8 cells per ml.<br>B cells were enriched by magnetic separation using anti-mouse CD19 microbeads (Miltenyi) following incubation of the cells with Fcgamma receptor IIB-blocking antibody.<br>Staining was performed on ice for 10 minutes.
Instrument	Cell separation by FACS was done at BD FACSAria II and Sony MA900 Multi-Application Cell Sorter. For cytometric analyses Canto II, Fortessa, Symphony (BD) or MACSQuant (Miltenyi) machines were used.
Software	Data were obtained using Sony MA900 software, BDs Diva or Miltenyi's MACSQuantify software and analyzed by FlowJo (version 9&10, TreeStar).
Cell population abundance	Purity and composition of sorted cells was assessed by cytometry directly after sort employing the same gating strategy used for gating. Purity of sorted cells was generally 95% or higher. In addition, the study did minimize effect of contamination by design, i.e. by single cell sequencing and/or a PCR step for amplification of Ig gene transcripts.
Gating strategy	Generally, antibody cell surface staining was performed on freshly isolated and unfixed primary cells. The gating strategy for quantification and charaterisation of isotype-switched memory B cells used was:<br>1) gate on lymphocytes, 2) exclude dead cells by propidium iodide or live/dead staining reagent, 3) 2D gate on surface switched Ig (e.g. IgG2b) positive cells against IgD, 4) gate on cells highly expressing CD19 and CD38 to exclude plasmablasts and recently activated B cells, 5) exclude cells positive for CD138, GL7, and IgM (combined to dump channel) to minimize inclusion of naive or germinal centre B cells and plasmablasts, 6) gate on small lymphocytes, and 7) exclude cell doublets.

☒ Tick this box to confirm that a figure exemplifying the gating strategy is provided in the Supplementary Information.
